# Causal association of physical activity with low back pain, intervertebral disc degeneration and sciatica: a two-sample mendelian randomization analysis study

**DOI:** 10.3389/fcell.2023.1260001

**Published:** 2023-11-09

**Authors:** Xiaoqing Guan, Ding Zhang, Fengyi Zhang, Yuan Zong, Hang Wang, Zhubin Shen, Fei Yin

**Affiliations:** Departments of Orthopedics Surgery, China-Japan Union Hospital of Jilin University, Changchun, China

**Keywords:** physical activity, low back pain, intervertebral disc degeneration, sciatica, mendelian randomization

## Abstract

**Objective:** Previous studies are insufficient to confirm a causal association between physical activity (PA) and low back pain (LBP), intervertebral disc degeneration (IDD), and sciatica. The present study used a two-sample Mendelian randomization (MR) analysis method to demonstrate whether or not there was a causal connection.

**Methods:** First, four PA phenotypes were selected [accelerometer-based PA (average acceleration), accelerometer-based PA (acceleration fraction >425 mg), self-reported moderate-to-vigorous PA, and self-reported vigorous PA], setting thresholds for single nucleotide polymorphisms (SNPs) significantly concerned with PA *p* < 5 × 10^−8^, linkage disequilibrium (LD) *r* ^2^ < 0.01, genetic distance >5,000 kb, and F-value >10. SNPs associated with the outcome and confounding factors were then excluded using the PhenoScanncer database. Finally, after coordinating the genetic instruments from genome-wide association studies (GWAS) effect alleles for exposure and outcomes, multiplicative random effects inverse variance weighting (IVW), MR-Egger, weighted median method (WMM), and weighted mode method were used to assess exposure-outcome causality and perform sensitivity analysis on the estimated results.

**Results:** The current study’s IVW findings revealed proof of a causal connection between PA and LBP. While there was a positive causal tie between accelerometer-based PA (acceleration fraction >425 mg) and LBP [OR: 1.818, 95% CI:1.129–2.926, *p* = 0.012], there was a negative causal link between accelerometer-based PA (average acceleration) and LBP [OR: 0.945, 95% CI: 0.909–0.984, *p* = 0.005]. However causal relationship between PA and IDD or sciatica was not found.

**Conclusion:** Increasing average PA but needing to avoid high-intensity PA may be an effective means of preventing low back pain. Although PA is not directly causally related to disc degeneration and sciatica, it can act through indirect pathways.

## 1 Introduction

Low back pain (LBP) is a high-incidence disease of the bony musculoskeletal system, which has a substantial economic burden on patients and society, and lumbar disc degeneration is one of the leading causes of its occurrence (Costăchescu et al., 2022; Mohd Isa et al., 2022). In a study, it was found that the prevalence of Intervertebral disc degeneration (IDD) was 71% in men and 77% in women under the age of 50, while the prevalence was as high as 90% in both men and women over the age of 50, and that lumbar disc degeneration was found to be significantly and positively associated with LBP (Teraguchi et al., 2014). In addition, degeneration of the lumbar intervertebral discs can also cause sciatica. L4, L5, S1, and S2 nerve roots are involved in the formation of the sciatic nerve, with compression of nerve roots in the L4-L5 and L5-S1 segments leading to sciatica being the most common (Ropper and Zafonte, 2015).

Previous epidemiological studies on risk factors for low back pain, disc degeneration, and sciatica suggest that various lifestyle-related factors may be associated with the development of these spinal disorders, such as age, gender, depression, isolation, education, alcohol consumption, smoking, obesity, and physical activity (PA) (Mikkelsson et al., 2006; Shiri et al., 2013; Shiri and Falah-Hassani, 2017; Yang and Haldeman, 2018). PA is broadly defined as energy-consuming musculoskeletal movements (Caspersen et al., 1985). Studies have shown that active PA can be effective in preventing and treating different chronic diseases (such as diabetes, cardiovascular disease, and cancer) and improving physical and mental function (Mok et al., 2019; Barker and Eickmeyer, 2020). In recent years, much discussion has been on the connection between PA and LBP. A recent cross-sectional study found that PA and prolonged sedentary behavior from childhood to adolescence increased the risk of LBP in adolescents and that early PA may be an essential factor in preventing LBP in adolescents (Lemes et al., 2022). In another study on PA and skeletal muscle pain in adolescents, similar conclusions were reached, in that moderate levels of PA reduced the risk of LBP, and endurance exercise was found to be associated with lower odds of LBP, the association that was particularly significant in girls (Guddal et al., 2017). Although these observational studies suggest a potential protective effect of PA on back pain, the causal relationship between the two needs to be further explored. The first thing to consider is a possible inverse cause-and-effect link between PA and back pain. Several studies have suggested that pain significantly contributes to reduced or impaired PA (Koho et al., 2011; Gremillion et al., 2022). Second, observational studies are difficult to avoid being confounded by many factors and producing false associations, such as age, gender, depression, loneliness, education level, alcohol consumption, smoking, and obesity. Third, most traditional observational studies use self-report assessments of PA, which are susceptible to recall and social desirability biases. A study of blue-collar workers found that self-reported sedentary time was underestimated, while moderate-to-vigorous PA time was overestimated (Gupta et al., 2018). Exploring the causes of such spinal disorders is necessary to help us prevent their occurrence and develop more effective treatments. However, traditional observational studies have inherent limitations such as confounding and reverse causation that may bias the results, while conducting randomized controlled trials (RCTs) is labor-intensive, time-consuming, and ethically constrained.

With the development of epidemiological studies, Mendelian randomization (MR) methods are increasingly used to estimate the causality of risk factors (exposures) on disease (outcomes). MR analysis requires the introduction of genetic variation as an instrumental variable (IV) to determine the link of causality between exposure and outcome (Smith and Ebrahim, 2003). The approach is also regarded as a natural RCT that eliminates confounding, reverse causation, and several other types of bias to evaluate the causal link between exposure and result since genetic variations are randomly allocated at conception and determined before starting (Smith and Ebrahim, 2004; Lawlor et al., 2008; Bahls et al., 2021). In spinal disorders, using MR methods has demonstrated a causal relationship between underlying exposure factors and pain, such as obesity (Zhou et al., 2021) and diabetes (Jin et al., 2023).

In order to provide a foundation for the prevention and treatment of these conditions, a two-sample MR analysis was employed in this study to determine whether there is a causal connection between PA and LBP, IDD, and sciatica.

## 2 Methods

**FIGURE 1:**
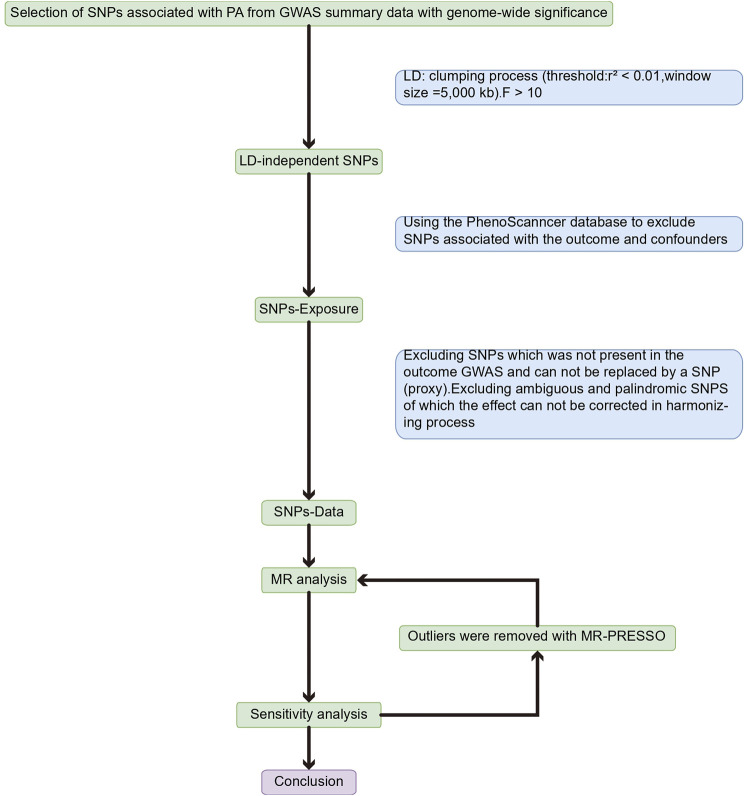
Flow chart about the analytical methods and the step-by-step MR analysis.

**FIGURE 2:**
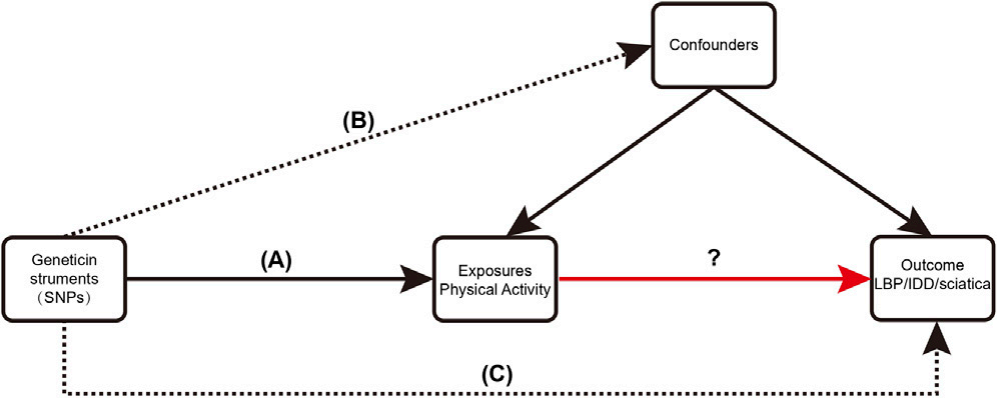
Three critical assumptions of the MR study. **(A)** The instrumental variables (IVs) must be associated with exposure (PA); **(B)** IVs must be independent of confounders; **(C)** IVs should not be directly associated with outcomes (LBP, IDD, sciatica).

An overview of the study design is shown in Figure 1. With the use of openly accessible data from the genetic instruments from genome-wide association studies (GWAS), we evaluated the causal connection between PA and three back diseases in this study using a two-sample MR strategy, followed by a sensitivity analysis to assess the stability of MR analysis results in the presence of violations of MR assumptions. The study was analyzed using previously published data and therefore did not require ethical approval. Using PA as the exposure factor and the presence of LBP, IDD, and sciatica as outcome variables, the potential causal association between exposure and outcome was analyzed using MR methods. The single nucleotide polymorphism (SNPs) involved in MR analysis as instrumental variables (IVs) in this study had to satisfy three core assumptions (Emdin et al., 2017): (1) SNPs were directly associated with PA; (2) SNPs were independent of any confounding factors; and (3) SNPs affected LBP, IDD, and sciatica through PA only (Figure 2).

### 2.1 Data source

PA can often be measured in terms of energy expenditure, which is described in units of metabolic equivalent (MET). The International Physical Activity Questionnaire (IPAQ) based on self-reporting and device measurements using wrist-worn triaxial accelerometers are two common methods of assessing energy expenditure (Xu et al., 2021). The GWAS for PA was derived from the United Kingdom Biobank (Klimentidis et al., 2018). A sizable prospective cohort study called the United Kingdom Biobank encompasses 400,000 individuals between the ages of 40 and 69 (Fry et al., 2017). Four phenotypes of PA were selected from this GWAS, specifically categorized into objective measures of accelerometer-based PA (average acceleration) and accelerometer-based PA (acceleration fraction >425 mg); subjective measures of self-reported moderate-to-vigorous PA, and self-reported vigorous PA (Hu et al., 2022). The researchers classified PA into average and vigorous (acceleration fraction >425 mg corresponding to an equivalent of vigorous physical activity) levels based on PA expressed by accelerometer measurements (Participants wearing Axivity AX3 wrist-worn accelerometers) (Klimentidis et al., 2018; Zhuo et al., 2021; Hu et al., 2022). Based on information from 377,234 people in the United Kingdom Biobank who filled out a touchscreen questionnaire on their PA (Similar to the International Physical Activity Questionnaire), PA was classified as moderate-to-vigorous and vigorous (Craig et al., 2003; Klimentidis et al., 2018; Hu et al., 2022). The four PAs have been widely used in both observational studies and Mendelian randomization studies, and although they are measured in different ways, they are all used in scientific studies by calculating metabolic equivalents reflecting the different strengths of PA, such as the studies between PA and ankylosing spondylitis (Hu et al., 2022) and osteoporosis (Xu et al., 2021). The results of applying these four types of PA for MR analysis are more easily comparable with the results of other studies, and the data are more readily available from a wide range of sources. A threshold of *p* < 5 × 10^−8^ was set for SNPs to be significantly associated with PA, [if too few SNPs were obtained for MR analysis, the threshold could be appropriately relaxed to *p* < 5 × 10^−7^ (Choi et al., 2019) ], and linkage disequilibrium (LD) *r* ^2^ < 0.01 and genetic distance >5000 kb were considered. The strength of the association between IVs and exposure factors was assessed by calculating the F statistic, and in general, a more significant F value indicated a stronger association between IVs and exposure, and the bias due to weak IVs was considered negligible when the mean F statistic was >10 (Burgess and Thompson, 2011).

The GWAS for LBP, IDD, and sciatica were all from the FinnGen consortium (https://www.finngen.fi/en) and included LBP (9,917 cases and 134,889 controls), IDD (15,565 cases and 134,889 controls), and sciatica (6,827 cases and 134,889 controls).

In addition, to further confirm SNPs as IVs, we used the PhenoScanncer database to exclude SNPs associated with LBP, IDD, and sciatica and their risk factors, like obesity, smoking, and alcohol consumption.

### 2.2 MR analysis

Multiplicative random effects inverse variance weighting (IVW), MR-Egger, weighted median method (WMM), and weighted mode were utilized to evaluate the exposure-outcome causality after coordinating the effect alleles for exposure and outcome. These four methods have different underlying assumptions regarding genetic variation as a valid IV. The Wald ratios computed for each SNP to provide an overall estimate of the influence of exposure on outcome are effectively weighted in the IVW estimate. Furthermore, the IVW assumes that all genetic variants are valid IVs. In other words, if there is no horizontal pleiotropy (genetic variants affect outcomes through pathways other than exposure), the causal impact estimate based on the approach is reliable (Burgess and Thompson, 2017). Although the IVW method specifically requires that genetic variants affect target outcomes only through exposure in the study, and studies have excluded known confounding SNPs to the extent possible, the prevalence of single genetic variants affecting multiple traits does not guarantee that all IVs are consistent with the hypothesis leading to horizontal pleiotropy and biasing estimates of effect values. In the MR-Egger approach (consisting of three components), it is assumed that the estimation precision of a single study is equal to the strength of a single IV, arguing that even if all genetic variation violates the IV hypothesis (100% null IVs), it gives consistent estimates of causal effects (Bowden et al., 2015). The validity control of IV is also relaxed in the WWM to give consistent estimates of causal effects even when the null IVs that violate the assumptions reach 50% (Bowden et al., 2016). The weighted mode approach groups SNPs with comparable causal estimates and evaluates the causal link between exposure and outcome using the greatest collection of SNPs. In other words, an unbiased causal estimate can be derived as long as the set of SNPs is valid IV (Hartwig et al., 2017).

### 2.3 Heterogeneity test and sensitivity analysis

Heterogeneity suggests either incorrect modeling assumptions or broken IV assumptions (Hemani et al., 2018). In this experiment, Cochran Q was applied to test the heterogeneity among SNPs in IVW assessment and if the heterogeneity *p* > 0.05 indicated no significant heterogeneity.

In order to identify and adjust for horizontal pleiotropic outliers, MR-PRESSO was utilized, which requires the InSIDE assumption (association of genetic variation with exposure is independent of the direct influence of genetic variation on the outcome) and that at least 50% of genetic variation be a valid IV (no horizontal pleiotropy) (Verbanck et al., 2018).

Potential horizontal pleiotropy was assessed using the MR-Egger regression intercept. The MR-Egger method is a weighted linear regression with the same intercept unconstrained as IVW, where the intercept represents the mean horizontal pleiotropy of genetic variance. If the MR-Egger regression intercept is different from zero (*p* < 0.05), it is evidence of horizontal pleiotropy (Bowden et al., 2015). Under the InSIDE assumption, the slope coefficient of MR-Egger regression provides a reliable estimate of the causative influence.

Leave-one-out analysis was performed in order to test whether MR causality estimates may be biased by a single SNP driven with significant levels of pleiotropy.

The statistical analysis of this study was based on the R software (version 4.0.2). The TwoSampleMR package was used to perform the MR analysis, which is publicly available for download on the GitHub website (https://mrcieu.github.io/TwoSampleMR/). The MRPRESSO package was applied to detect horizontal pleiotropy. The forestplot package, as well as the TwoSampleMR package were applied to plot forest plots, scatter plots, and funnel plots related to MR analysis. TwoSampleMR version 0.5.6; MRPRESSO 1.0; Forestplot 2.0.1.

## 3 Results

The SNPs used in this study all met the threshold of significant association with exposure *p* < 5 × 10^−8^ [except for SNPs associated with accelerometer-based PA (average acceleration) with a relaxed threshold of *p* < 5 × 10^−7^] (LD) *r* ^2^ < 0.01, and genetic distance >5000 kb. Furthermore, the F-value is in the range of 25.35917–51.82382, all >10, with the bias from weak IVs largely ignored. We used the PhenoScanncer database to identify SNPs associated with outcomes or potential confounders to be excluded. The removed SNPs’ specifics are displayed in Supplementary Table S1. IVW, MR-Egger, WMM, and weighted mode were used to derive the results of the relationship between PA and the three back diseases. Supplementary Table S2 provides specifics on the SNPs utilized for the MR analysis. The results of the sensitivity analysis confirmed the stability of the MR analysis results. Firstly, the Cochran Q test for heterogeneity was applied, and there was no evidence of heterogeneity in the study’s findings, according to the *p*-value >0.05 (Supplementary Figures S1–S3). Then the *p* values for the MR-Egger regression intercept were all >0.05, indicating no horizontal pleiotropy to bias the results. No outliers were found for MR-PRESSO and *p* ≥ 0.05 for each group for the horizontal pleiotropy test (except self-reported moderate-to-vigorous PA exposure, LBP for the outcome group, *p* = 0.038). The results of the final Leave-one-out analysis also did not find SNPs that caused significant bias to the results (Supplementary Figures S4–S6). Details of the results are shown in Supplementary Table S3–S5.

### 3.1 PA and LBP

**FIGURE 3:**
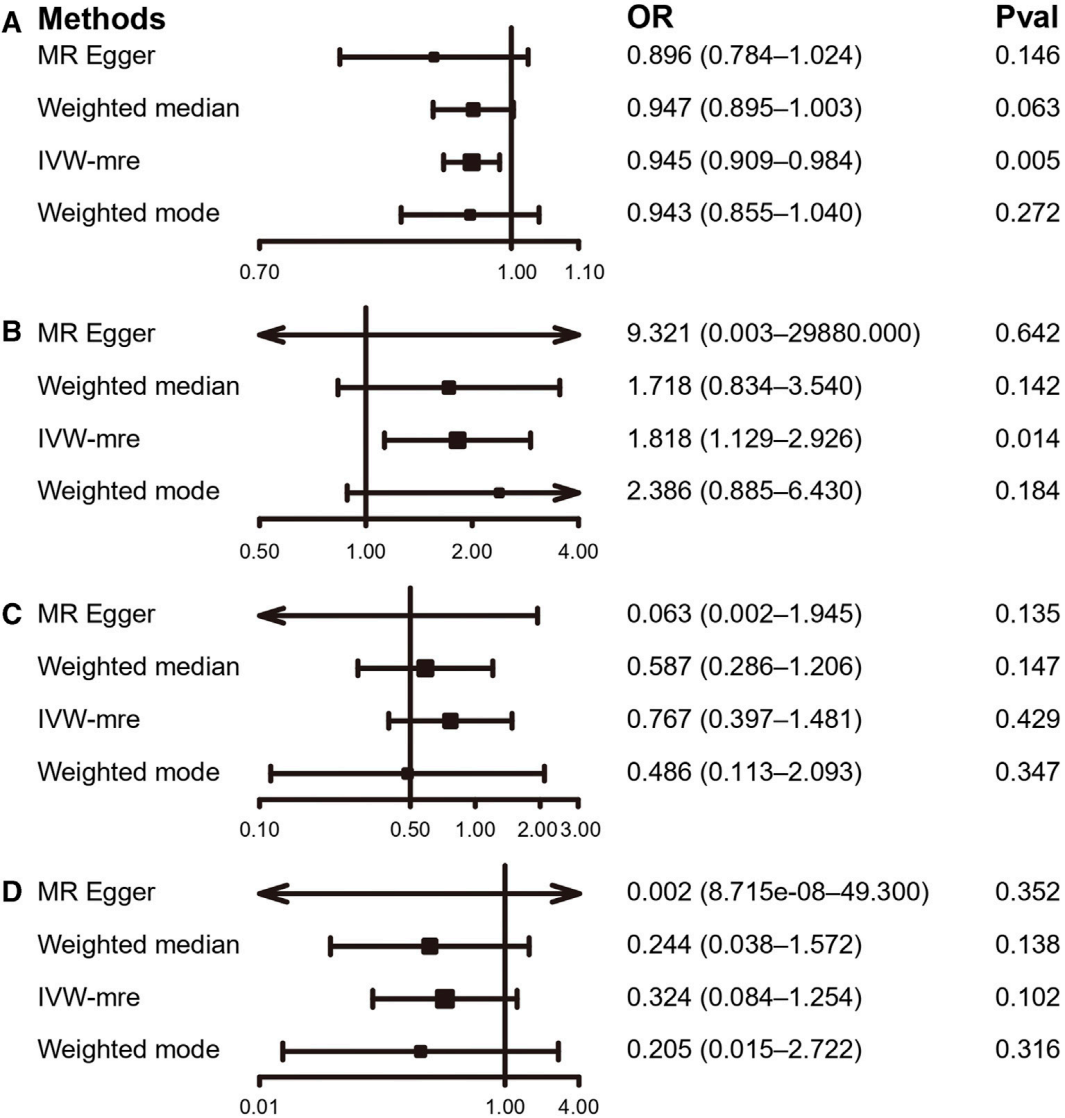
MR results of the association between PA and LBP. **(A)** accelerometer-based PA (average acceleration); **(B)** accelerometer-based PA (acceleration fraction >425 mg); **(C)** self-reported moderate-to-vigorous PA; **(D)** self-reported vigorous PA. OR, odds ratio; CI, confidence interval; IVW-mre, multiplicative random effects inverse variance weighting.

**FIGURE 4:**
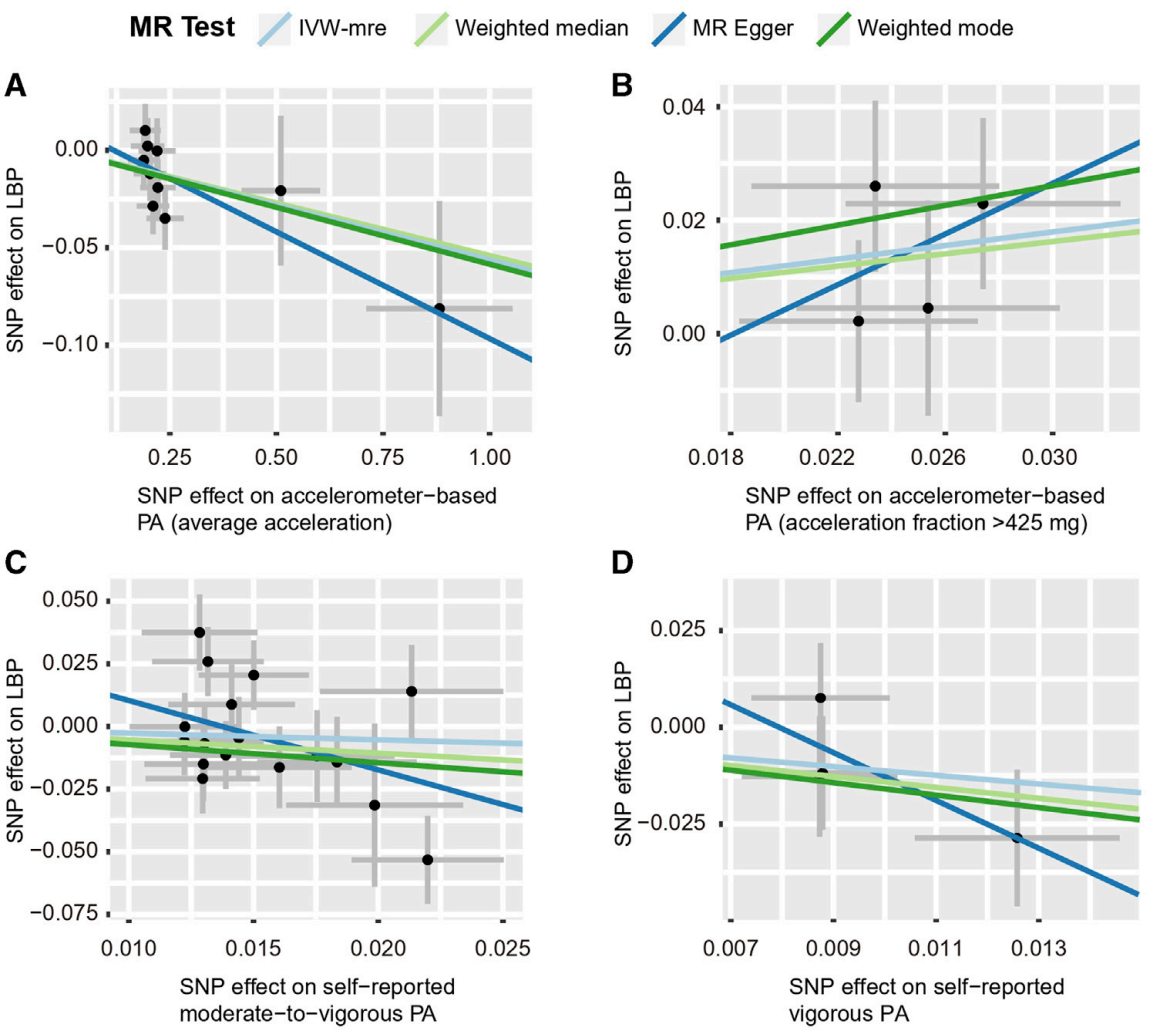
Scatter plots for MR analyses of the causal effect of PA on LBP. **(A)** accelerometer-based PA (average acceleration); **(B)** accelerometer-based PA (acceleration fraction >425 mg); **(C)** self-reported moderate-to-vigorous PA; **(D)** self-reported vigorous PA. Analyses were conducted using the conventional IVW, WMM, MR-Egger, and weighted mode methods. The slope of each line corresponds to the estimated MR effect per method. IVW-mre, multiplicative random effects inverse variance weighting.

Using the IVW estimate, the current study discovered evidence of a causal connection between accelerometer-based PA and LBP. While there was a negative causative association between accelerometer-based PA (average acceleration) and LBP [OR: 0.945, 95% CI: 0.909–0.984, *p* = 0.005], there was a positive causal link between accelerometer-based PA (acceleration fraction >425 mg) and LBP [OR: 1.818, 95% CI:1.129–2.926, *p* = 0.014]. Although MR-Egger, WMM, and weighted model assessment results may have *p*-values greater than 0.05, the OR values were consistent with the IVW trend, so the IVW estimates were still plausible (Figures 3A, B; Figures 4A, B; Supplementary Table S3 and Supplementary Figures S7A, B). There was no proof that self-reported PA and LBP were related causally. IVW estimates suggested either self-reported moderate-to-vigorous PA [OR: 0.767, 95% CI: 0.397–1.481, *p* = 0.429] or self-reported vigorous PA [OR: 0.324, 95% CI. 0.084–1.254, *p* = 0.103]. MR-Egger, WMM, and weighted mode are consistent with the IVW estimation results (Figures 3C, D; Figures 4C, D); Supplementary Table S3 and Supplementary Figures S7C, D).

### 3.2 PA and IDD

**FIGURE 5:**
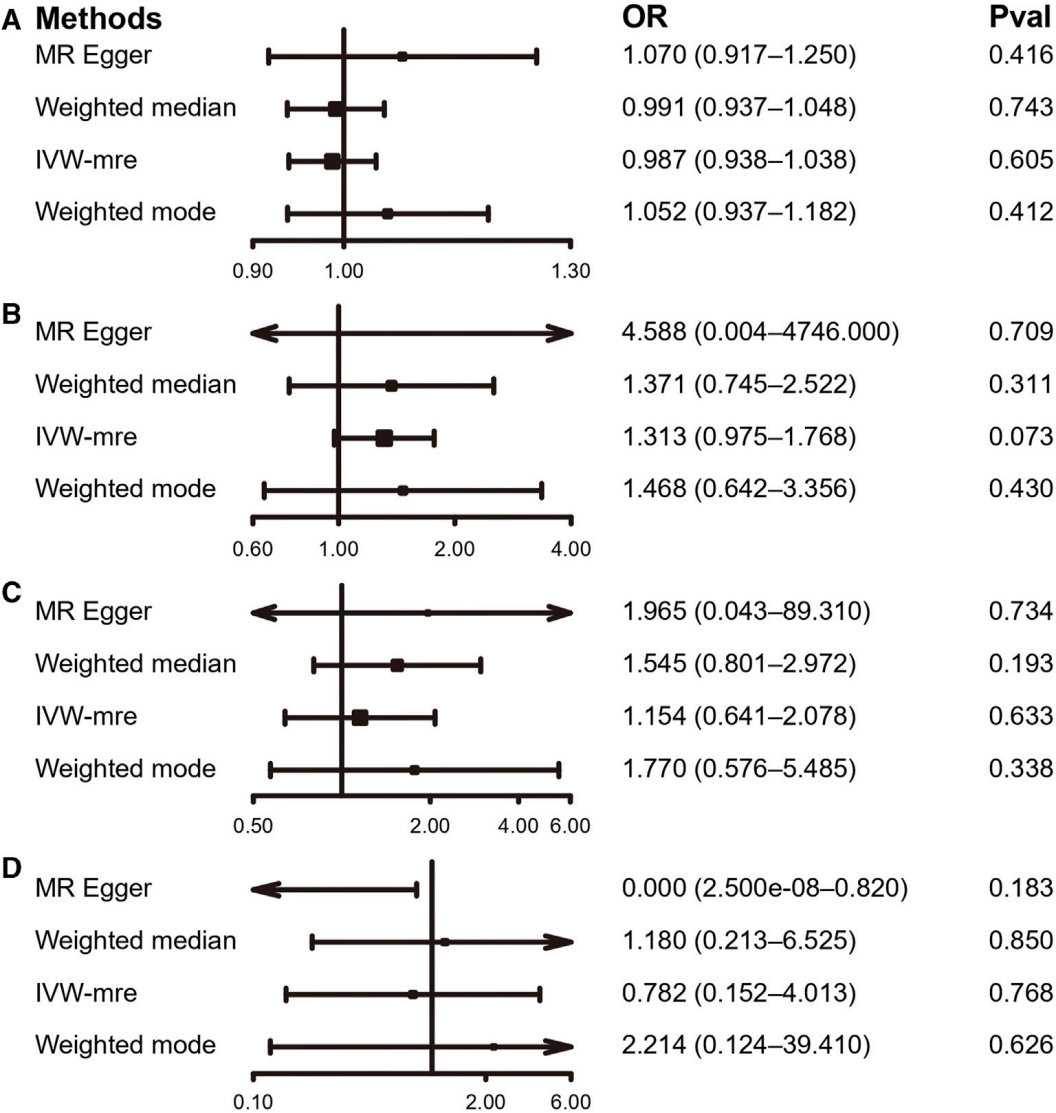
MR results of the association between PA and IDD. **(A)** accelerometer-based PA (average acceleration); **(B)** accelerometer-based PA (acceleration fraction >425 mg); **(C)** self-reported moderate-to-vigorous PA; **(D)** self-reported vigorous PA. OR, odds ratio; CI, confidence interval; IVW-mre, multiplicative random effects inverse variance weighting.

**FIGURE 6:**
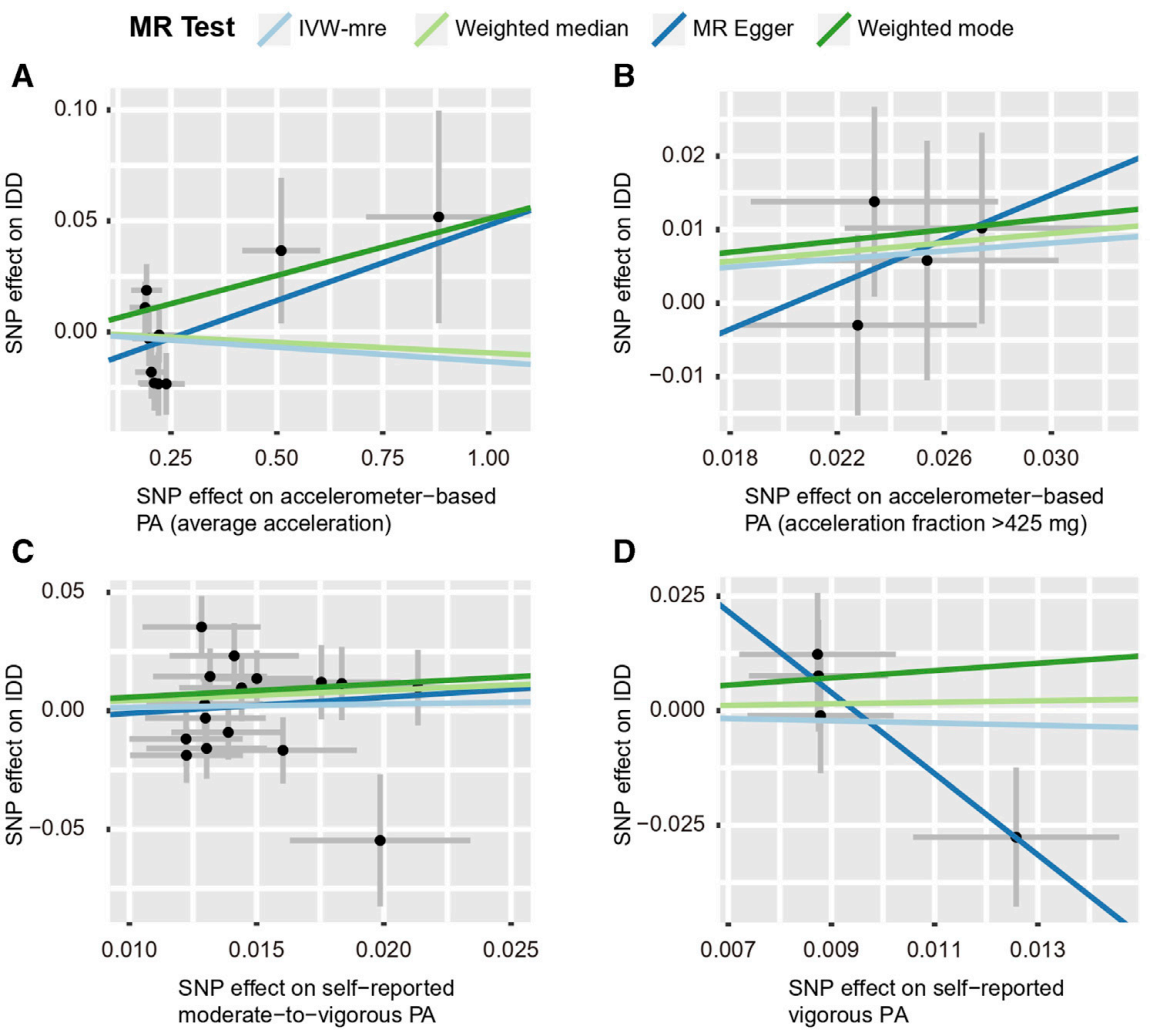
Scatter plots for MR analyses of the causal effect of PA on IDD. **(A)** accelerometer-based PA (average acceleration); **(B)** accelerometer-based PA (acceleration fraction >425 mg); **(C)** self-reported moderate-to-vigorous PA; **(D)** self-reported vigorous PA. Analyses were conducted using the conventional IVW, WMM, MR-Egger, and weighted mode methods. The slope of each line corresponds to the estimated MR effect per method. IVW-mre, multiplicative random effects inverse variance weighting.

By using the IVW estimate, this study was unable to identify any data supporting a causal link between PA and IDD. Accelerometer-based PA (average acceleration) [OR: 0.987, 95% CI: 0.938–1.038, *p* = 0.605], accelerometer-based PA (acceleration fraction >425 mg) [OR: 1.313, 95% CI:0.975–1.768, *p* = 0.073], self-reported moderate-to-vigorous PA [OR: 1.154, 95% CI:0.641–2.078, *p* = 0.633], self-reported vigorous PA [OR: 0.782, 95% CI:0.152–4.013, *p* = 0.768], all four PA above suggested no causal relationship with the IVW estimates of IDD. Similarly, the IVW predicted findings were compatible with the MR-Egger, WMM, and weighted mode (Figures 5, 6; Supplementary Table S4 and Supplementary Figure S8).

### 3.3 PA and sciatica

**FIGURE 7:**
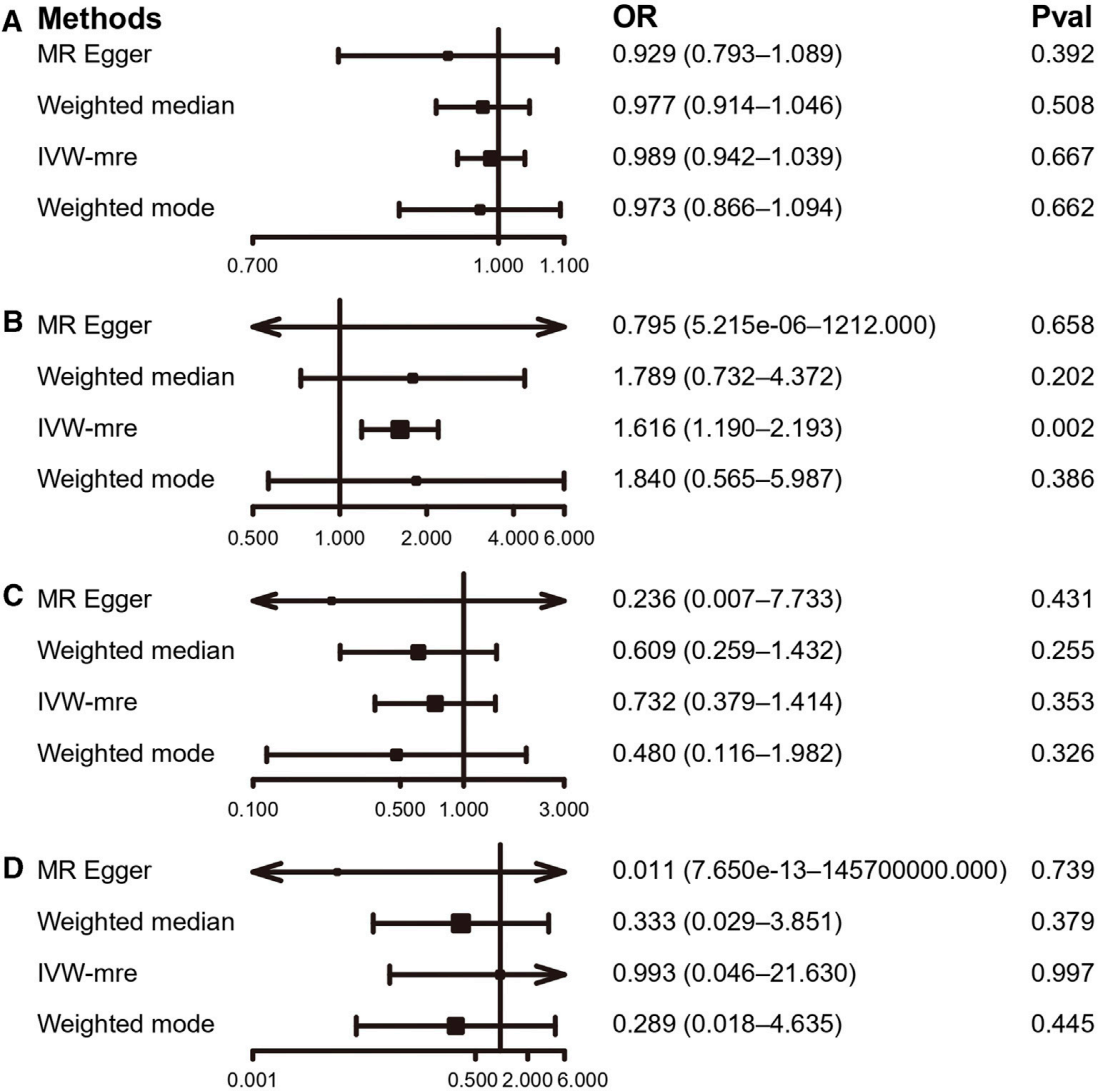
MR results of the association between PA and sciatica. **(A)** accelerometer-based PA (average acceleration); **(B)** accelerometer-based PA (acceleration fraction >425 mg); **(C)** self-reported moderate-to-vigorous PA; **(D)** self-reported vigorous PA. OR, odds ratio; CI, confidence interval; IVW-mre, multiplicative random effects inverse variance weighting.

**FIGURE 8:**
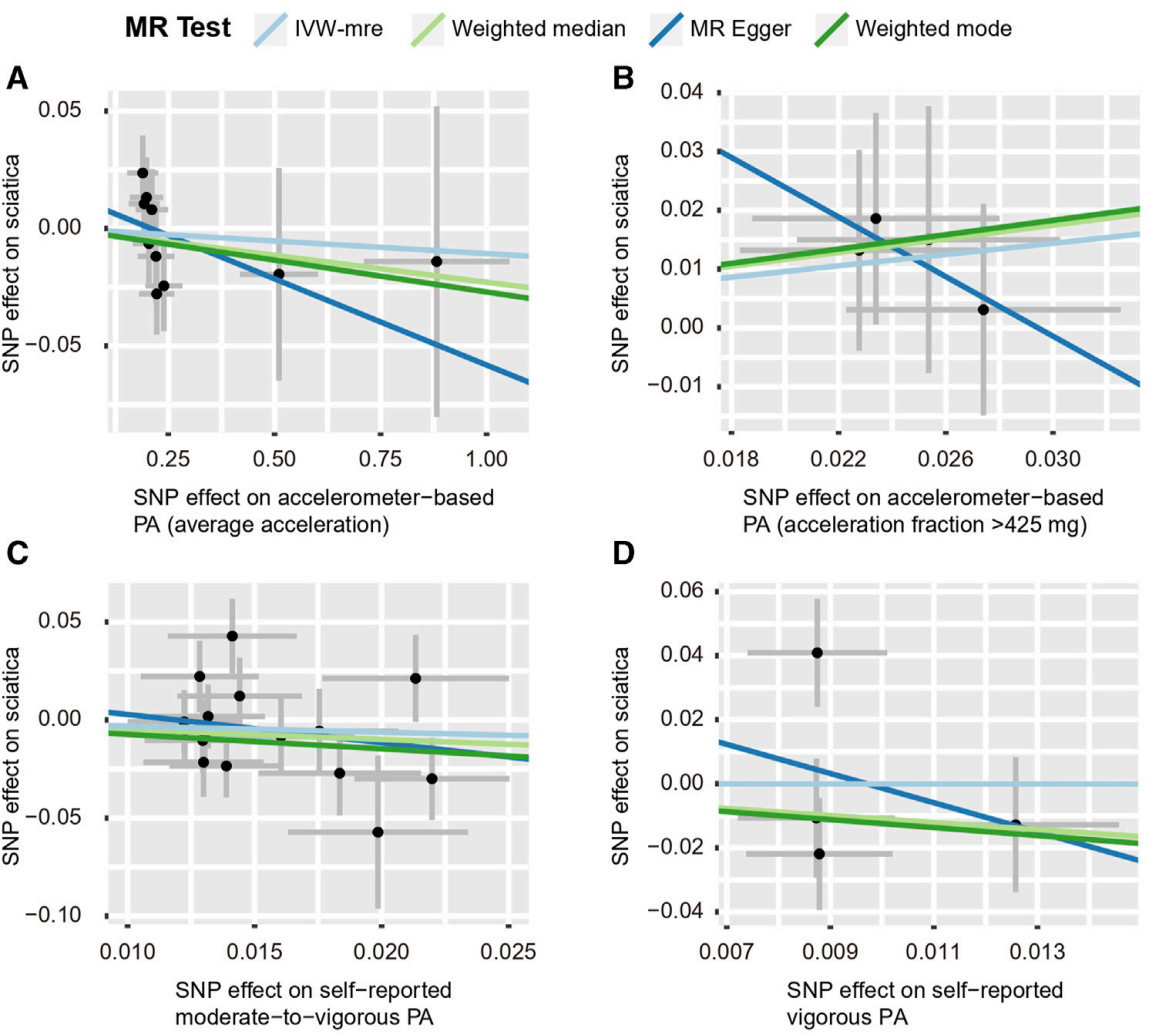
Scatter plots for MR analyses of the causal effect of PA on sciatica. **(A)** accelerometer-based PA (average acceleration); **(B)** accelerometer-based PA (acceleration fraction >425 mg); **(C)** self-reported moderate-to-vigorous PA; **(D)** self-reported vigorous PA. Analyses were conducted using the conventional IVW, WMM, MR-Egger, and weighted mode methods. The slope of each line corresponds to the estimated MR effect per method. IVW-mre, multiplicative random effects inverse variance weighting.

According to the IVW estimate, our present study did not find any proof of a causal connection between PA and sciatica. Although the accelerometer-based PA (acceleration fraction >425 mg) [OR: 1.616, 95% CI:1.19–2.193, *p* = 0.002] had a *p*-value <0.05 with the results of IVW analysis for sciatica. However, the MR-Egger [OR: 0.080, 95% CI: 5.215 × 10^-6^–1,212.000, *p* = 0.658] trended opposite to the OR of the IVW estimate, so we also considered the result negative. The IVW estimates between PA and sciatica in the other three groups, accelerometer-based PA (average acceleration) [OR: 0.989, 95% CI: 0.942–1.039, *p* = 0.667], self-reported moderate-to-vigorous PA [OR: 0.732, 95% CI: 0.379–1.414, *p* = 0.353], and self-reported vigorous PA [OR: 0.993, 95% CI: 0.046–21.630, *p* = 0.997], all with *p*-values greater than 0.05, similar MR-Egger, weighted median, and weighted mode agreed with the IVW projections as well. (Figures 7, 8; Supplementary Table S5 and Supplementary Figure S9).

## 4 Discussion

In order to thoroughly assess whether PA directly impacts the incidence of three back diseases, we used a two-sample MR technique. We discovered a causal association between PA and LBP but no conclusive evidence to support the causative role of genetically predicted PA on the risk of IDD and sciatica. There was no direct link between self-reported moderate to vigorous PA and vigorous PA and LBP. However, average PA by accelerometer reduced the risk of LBP, whereas strong PA increased the risk. To our knowledge, this study presents the first thorough MR evaluation of the relationship between PA and LBP risk. In the current investigation, we were able to firmly establish causality aside from bias owing to superior study design by using MR approaches and differentiating across PA of various intensities and measures (four PA phenotypes in all). The results of our study are significant in that they give mechanical context to the observed relationships from prior investigations, demonstrating the connection between PA and LBP.

From our findings that accelerometer-based PA (average acceleration) lowers the danger of LBP, the results reflect that average level PA is beneficial in preventing LBP, similar to most observational studies that have found that PA reduces LBP. Results of a cross-sectional health survey conducted among the general population aged 50 years or older in Korea found that a lower incidence of LBP was highly connected with walking more than 3 days per week for more than 30 min while exercising more than 1 h per day and more than five times per week showed a more significant negative association with LBP (Park et al., 2019). Recent literature on observational studies showed a negative association between PA and LBP and that a low prevalence of LBP was correlated with moderate activity levels (Alzahrani et al., 2019). Similarly, literature involving 36 prospective cohort studies found that PA in spare time may be moderately protective against the development of frequent or chronic LBP (Shiri and Falah-Hassani, 2017). On the other hand, accelerometer-based PA (acceleration fraction >425 mg) increased the likelihood of LBP, suggesting that high-intensity PA contributes to the development of LBP. A cross-sectional study on LBP in Swedish adolescents came to similar conclusions: high-intensity exercise was linked to LBP risk, LBP duration, and LBP-related impairment (Sundell et al., 2019).

LBP can be influenced by a variety of factors, both local and systemic, such as structural failure of musculoskeletal tissues, inflammatory and immune responses, emotional state, and behavioral and environmental factors (Vlaeyen et al., 2018). Regular PA can significantly improve the health of the musculoskeletal system by modulating bone and muscle factors. For bone, PA promotes osteogenesis by increasing Osteocalcin (OCN), Osteoprotegerin (OPG), and Fibroblast Growth Factor 23 (FGF23), decreases Receptor Activator of Nuclear Factor Kappa B (RANK)/RANK Ligand (RANKL) and sclerostin thereby inhibiting osteoblastogenesis (Hermann et al., 2020; Cariati et al., 2021); for muscle, PA increases muscle mass and strength by stimulating muscle tissue to produce Interleukins (IL-6, IL-7, IL-8, IL-10, and IL-15), irisin, and Beta-Aminoisobutyric Acid (BAIBA) while inhibiting myostatin secretion (Cariati et al., 2021). Previous studies have shown that PA can affect the expression of inflammatory factors (e.g., IL-6 and IL-10) in the serum of the elderly (Jankord and Jemiolo, 2004). Moreover, current studies have discovered that local immune reactions are related to aching muscles and that regular PA increases the percentage of skeletal muscle regulatory macrophages (M2, which secrete anti-inflammatory cytokines) and increases IL-10 (an anti-inflammatory factor) in mice to prevent chronic pain (Leung et al., 2016; Lesnak et al., 2023). In addition, there is a balance between inhibition and excitation in the central nervous system, and PA may upset this balance, thereby promoting analgesia or exacerbating pain. Regular exercise promotes pain relief characterized by reduced N-Methyl-D-aspartate (NMDA) receptor phosphorylation, and it was further found that regular exercise increased serotonin levels by decreasing the expression of the serotonin transporter and increased opioids in the central inhibitory pathway, suggesting that exercise utilizes our endogenous inhibitory system to reduce pain (Lima et al., 2017). The possibility that psychological factors may trigger or exacerbate low back pain should not be ignored. In a study on the causes of lower back pain, 3.1% of people cited psychological factors as the main determinants of low back pain (Knezevic et al., 2021); similarly, a study has found that low back pain is linked to depression and stress, and improving mental health may be a key issue in preventing musculoskeletal pain (Diepenmaat et al., 2006). Physical activity can not only strengthen the musculoskeletal system and reduce systemic inflammation and pain, but also improve mental health and quality of life, but it needs to be tailored to individual interests, needs, and abilities to achieve greater benefits (Paley and Johnson, 2016). This study clarifies the causal relationship between PA and LBP and lays the foundation for further exploration of complex molecular biological mechanisms.

Notably, our results show that self-reported moderate-to-vigorous PA and self-reported vigorous PA are unrelated to LBP. Equally, 12-year longitudinal research found no indication that exercise intensity altered the nature of the connection between PA and LBP (Cunningham et al., 2022). The reason for this divergence is related to the limitations of self-report measures. Participants’ emotions, memories, and social desirability biases could impact their responses to self-report, which may diminish the “true” association with outcomes. Nevertheless, this does not negate the usefulness of self-report assessments; objective measures are more accurate and reliable than self-report measures, so it is essential to employ objective measures to validate their findings (Choi et al., 2019; Baumeister et al., 2020; Meisinger et al., 2020; Sun et al., 2021). It should be highlighted that the prior publications’ study designs, which were mostly observational and could not completely rule out the influence of confounders or reverse causation, may cause disparate results.

Although the results in our study, whether using different PA evaluation methods or different intensities of PA, suggest no causal relationship between PA and either IDD or sciatica, this does not negate the existence of an association between PA and intervertebral discs. A 14-year cross-sectional case-control study found that decreased physical activity was associated with increased disc degeneration in the thoracolumbar spine (*p* < 0.05). The investigators quantified thoracic and lumbar disc degeneration by collecting questionnaire and MRI data using the Pfirrmann score, which showed that disc degeneration was significantly more pronounced in patients with irregular activity <1 h compared to those with regular activity ≥1 h per week (*p* < 0.01), and in patients with no activity compared to those with regular activity ≥2 h per week (*p* < 0.001) (Maurer et al., 2020). A meta-analysis that included four Finnish prospective cohort studies, including 34,589 participants and 1,259 hospitalized patients with sciatica, showed that walking or cycling to work reduced the risk of hospitalization for sciatica by 33% (95% CI 4%–53%) (Shiri et al., 2017). Similarly, a cross-sectional study involving 13,095 participants found that leisure-time physical activity appeared to protect men from sciatica, with findings showing that physical activity during leisure time in men reduced the risk of sciatica (0.74; 0.55–1.00) and was more significant in white-collar occupations (0.38; 0.18–0.88) (Euro et al., 2018). According to previous literature, different types of loads produce different effects on the intervertebral disc, such as dynamic loads, axial loads, static loads, torsional loads, and impact loads (Belavý et al., 2016; Ravalli and Musumeci, 2022). For example, a dynamic load of 0.2–0.8 MPa on the disc, which produces an intradiscal pressure of 0.3–1.2 MPa, is considered the optimal amount of load, and walking and running fall just within this load amplitude window (Belavý et al., 2017), while different types of PA will exert different types of loads on the intervertebral disc. Not only that, but intervertebral discs rely heavily on the diffusion of nutrient exchange through the endplates, and it has been found that different loads can affect the rate at which small solutes penetrate into the discs (Belavý et al., 2016). PA has been found to increase glycosaminoglycan concentrations in the intervertebral discs, and this relationship is related to training load (Ueta et al., 2018). In addition, obesity increases disc burden and is causally related to the risk of IDD, sciatica, and LBP (Zhou et al., 2021), while PA contributes to weight loss and weight maintenance after weight loss (Oppert et al., 2021). A Finnish study found that serum total cholesterol, LDL, cholesterol, and triglyceride levels were positively associated with the prevalence of sciatica in men and that PA was an essential factor affecting lipids (Heuch et al., 2010; Ou et al., 2017). In animal genetic experiments, the researchers found that PA reduced overall DNA methylation in healthy and pathologic intervertebral discs in mice and found that the effects of PA on mRNA expression of Dnmt3a, Mbd2b, and Tet1 were sex-specific (Kawarai et al., 2021).

Advantages. 1. The experimental design of MR reduces the potential for confounding or reverse causality compared to observational studies. Because it is sometimes difficult to determine whether PA precedes the onset of LBP in clinical work, whereas genotype is fixed to precede disease onset at conception, the MR approach minimizes the effect of reverse causality. Obesity (BMI) has been linked in certain studies to an increased incidence of LBP, IDD, and sciatica (Zhou et al., 2021). Another study found that current or former drinkers, obese or overweight individuals had higher odds of developing LBP (Yang and Haldeman, 2018). Therefore, our study excluded SNPs associated with confounding factors to reduce their bias on the assessment results. 2. By using a two-sample MR approach, we were able to test the impact of PA in large LBP (9,917 cases and 134,889 controls), IDD (15,565 cases and 134,889 controls), and sciatica (6,827 cases and 134,889 controls) cohort. This two-sample MR approach can have statistical power comparable to using individual-level data even when the necessary assumptions are not fully satisfied (Burgess et al., 2013), yet few cohorts have accumulated such a large number of LBP cases and controls. 3. Understanding the affiliation between PA and the three back illnesses would help public health strategies for early prevention and prompt intervention, which are essential given the high prevalence and disability rates of LBP, IDD, and sciatica in the population.

Disadvantages. 1. To avoid population stratification bias, the patients enrolled were from a European population, while in other populations, the causal relationship between PA and LBP, sciatica, and IDD is unclear. 2. Although we took multiple steps to check for pleiotropy, there could still be potential bias due to pleiotropy as the exact function of most SNPs is unknown. 3. There may be overlap in the cohorts, and the ideal situation for two samples of MR is no overlap between exposure and outcome. However, complete non-overlap is challenging to achieve using publicly available pooled data. 4. We cannot completely rule out the possibility that population stratification would impact the findings. 5. We could not look into possible correlations with u- or j-types, which hinders us from stating quantitatively how significantly more harmful excess PA is than moderate PA. 6. In the sensitivity analysis, the MR-Egger regression intercept did not find horizontal pleiotropy, and the IVW results were stable; neither the Leave-one-out analysis nor MR-PRESSO found a single SNP with significant horizontal pleiotropy. However, horizontal pleiotropy was found when using MR-PRESSO to examine the IVW results of this study with self-reported moderate-to-vigorous PA as the exposure and LBP as the outcome group. Thus, the stability of the results of this group needs to be further investigated. 7. Even though the GWAS, which has the most complete information and the largest sample size at present, was chosen for MR analysis in this study, the study will inevitably be limited by the bias generated by the experimental design and statistical methods of the original study.

## 5 Conclusion

The conclusions drawn from the above research findings demonstrate a causal connection between PA and the possibility of LBP. Average PA can reduce the risk of LBP, while high-intensity PA increases the risk of LBP, suggesting that increasing average PA but avoiding high-intensity PA may effectively prevent LBP. Our scientific findings are to be validated in sizable cohort prospective studies and should be viewed as proof of concept.

## Figures and Tables

**Table:** 

**PA**	physical activity
**LBP**	low back pain
**IDD**	intervertebral disc degeneration
**MR**	Mendelian randomization
**SNPs**	single nucleotide polymorphisms
**LD**	linkage disequilibrium
**IVW**	inverse variance weighting
**IVW-mre/IVW (mre)**	multiplicative random effects inverse variance weighting
**WMM**	weighted median method
**RCT**	randomized controlled trial
**GWAS**	genetic instruments from genome-wide association studies
**IVs**	instrumental variables
**MR-PRESSO**	MR-Pleiotropy RESidual Sum and Outlier method
**OR**	odds ratio
**CI**	confidence interval
**Se**	standard error
**Df**	degree of freedom

## Data Availability

Publicly available datasets were analyzed in this study. This data can be found here: https://www.finngen.fi/en https://www.ukbiobank.ac.uk/.
