# Supplementary material for: Causal association of physical activity with low back pain, intervertebral disc degeneration and sciatica: a two-sample mendelian randomization analysis study
Source: Front Cell Dev Biol. 2023 Nov 9;11:1260001. doi: 10.3389/fcell.2023.1260001 (PMC10665496; doi:10.3389/fcell.2023.1260001)
Supplement: Supplementary file 4 [file DataSheet4.pdf]

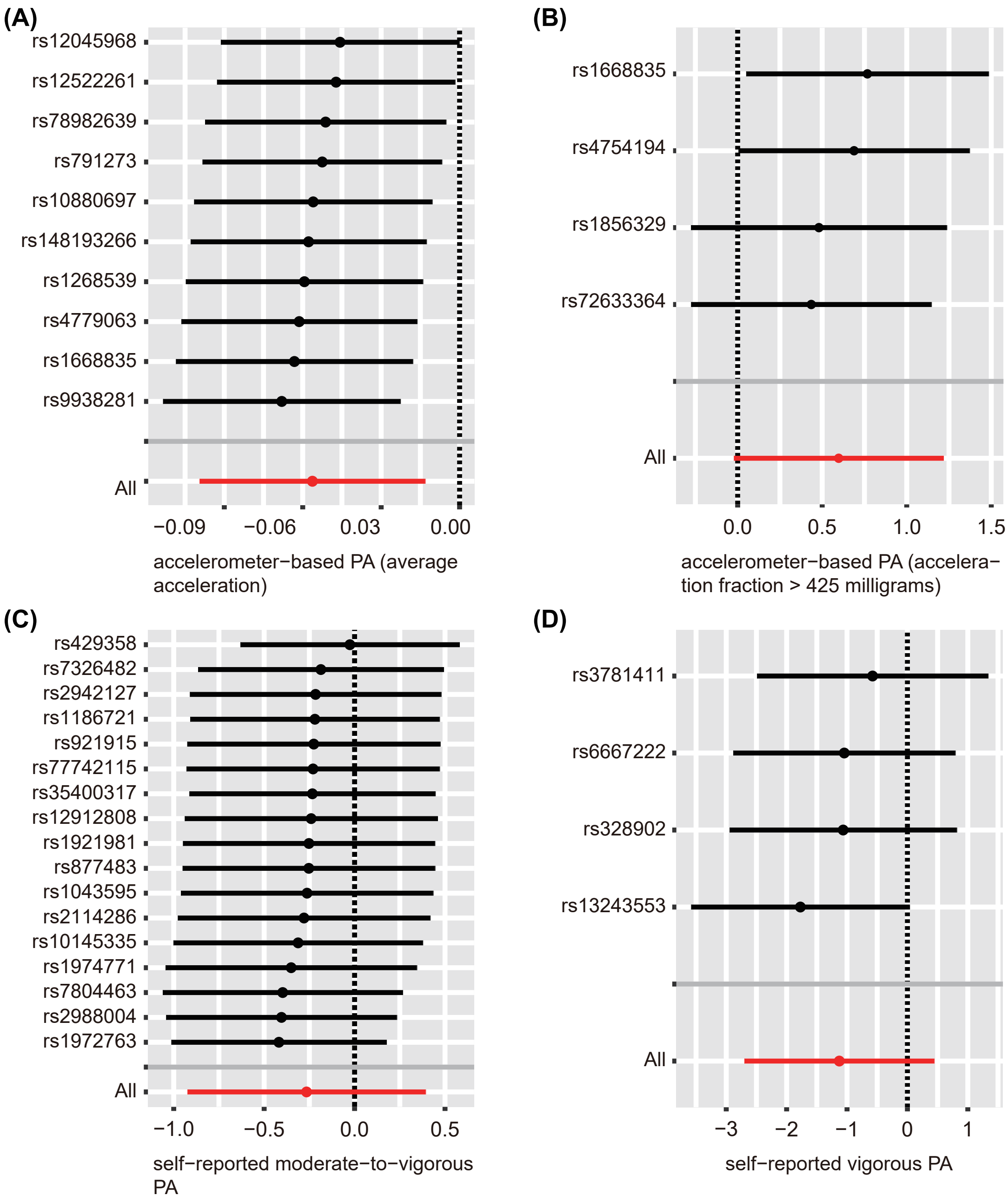

Figure S4: Leave-one-out sensitivity analysis of single SNP for LBP. (A) accelerometer-based PA (average acceleration); (B) accelerometer-based PA (acceleration fraction > 425 milligrams); (C) self-reported moderate-to-vigorous PA; (D) self-reported vigorous PA. Leave-one-out sensitivity analysis is performed to ascertain if a single SNP disproportionately influences an association for LBP. Each black point in the forest plot represents the MR analysis (using IVW) excluding that particular SNP. The overall analysis, including all SNPs, is also shown for comparison..
