# Supplementary material for: Causal association of physical activity with low back pain, intervertebral disc degeneration and sciatica: a two-sample mendelian randomization analysis study
Source: Front Cell Dev Biol. 2023 Nov 9;11:1260001. doi: 10.3389/fcell.2023.1260001 (PMC10665496; doi:10.3389/fcell.2023.1260001)
Supplement: Supplementary file 7 [file Table1.DOCX]

**Supplementary Table: Deleted SNPs associated with confounders**

|  | SNP | Reason for deletion |
| --- | --- | --- |
| Average accelerometer-based PA | | |
|  | rs34517439 | fat/ BMI |
|  | rs11012732 | fat/ BMI |
|  | rs59499656 | fat/ BMI |
|  | rs6775319 | fat/ BMI |
|  | rs9293503 | fat/ BMI |
|  | rs945890 | fat/ BMI |
|  | rs1550435 | fat/ BMI |
|  | rs55938136 | Alcohol intake frequency |
|  | rs56194509 | Alcohol intake frequency |
| Average accelerometer-based PA (fraction of accelerations >425 milligravities) | | |
|  | rs62443625 | fat/ BMI |
|  | rs743580 | fat/ BMI |
|  | rs80028338 | fat/ BMI |
| Self-reported moderate-to-vigorous PA | | |
|  | rs2035562 | fat/ BMI |
|  | rs2854277 | fat/ BMI，Diabetes |
|  | rs4886868 | fat/ BMI |
| Self-reported vigorous PA | | |
|  | rs1248860 | fat/ BMI |
|  | rs13212652 | fat/ BMI |
|  | rs559411160 | fat/ BMI，diabetes |
|  | rs9276758 | diabetes |
|  | rs2764261 | fat/ BMI |

SNP: Single Nucleotide Polymorphism; BMI: body mass index
