# Supplementary material for: Causal association of physical activity with low back pain, intervertebral disc degeneration and sciatica: a two-sample mendelian randomization analysis study
Source: Front Cell Dev Biol. 2023 Nov 9;11:1260001. doi: 10.3389/fcell.2023.1260001 (PMC10665496; doi:10.3389/fcell.2023.1260001)
Supplement: Supplementary file 8 [file DataSheet3.pdf]

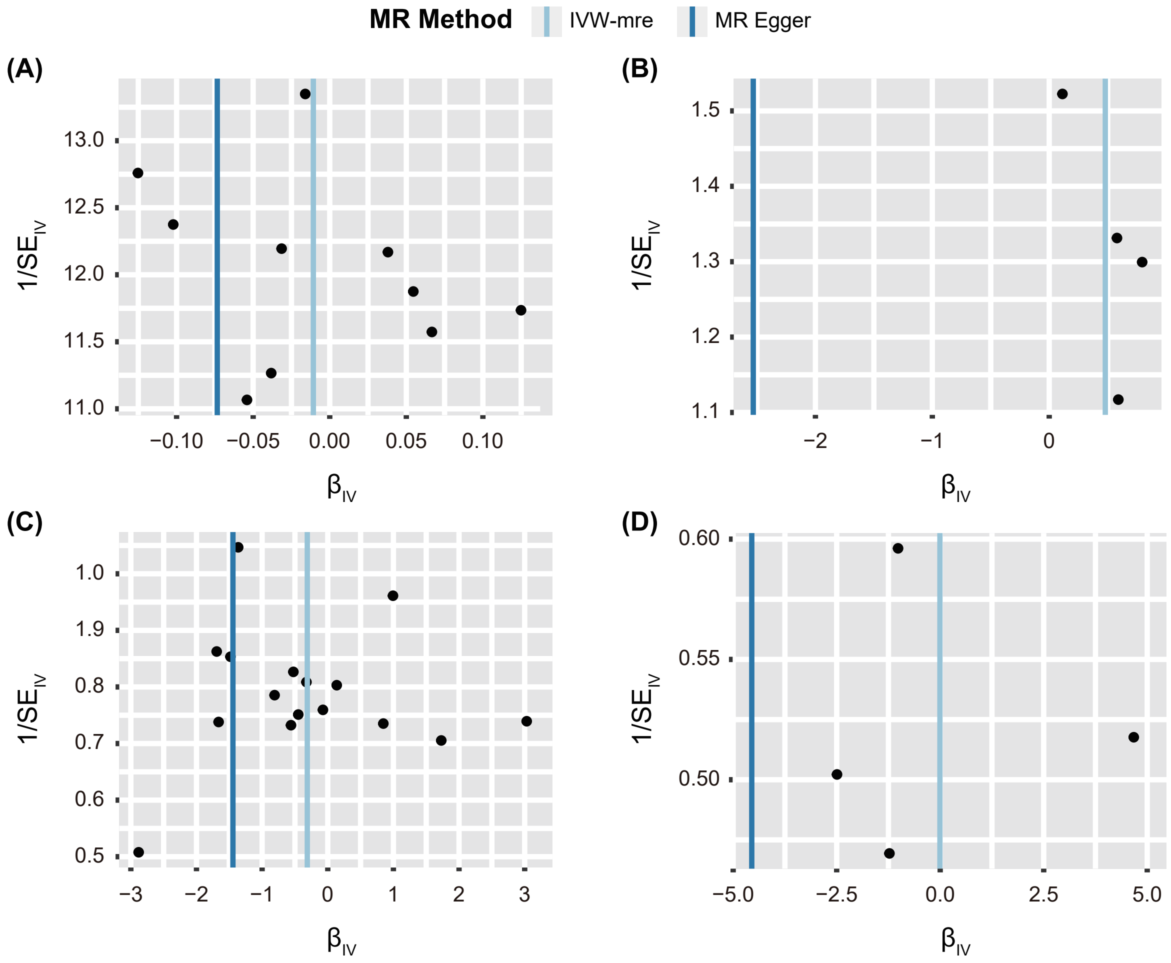

Figure S3: Funnel plots of the genetic associations between PA and sciatica. (A) accelerometer-based PA (average acceleration); (B) accelerometer-based PA (acceleration fraction > 425 milligrams); (C) self-reported moderate-to-vigorous PA; (D) self-reported vigorous PA. The light blue line represents the IVW estimate, and the dark blue line represents the MR‐Egger estimate. IVW-mre, multiplicative random effects inverse variance weighting.
