# Supplementary material for: Causal association of physical activity with low back pain, intervertebral disc degeneration and sciatica: a two-sample mendelian randomization analysis study
Source: Front Cell Dev Biol. 2023 Nov 9;11:1260001. doi: 10.3389/fcell.2023.1260001 (PMC10665496; doi:10.3389/fcell.2023.1260001)
Supplement: Supplementary file 10 [file Table5.DOCX]

Table S5 MR estimates from different methods of assessing the causal effect of PAs on sciatica

| PAs | No. of  SNP | IVW | | | | WMM | | MR-Egger | | | | | | Weighted mode | | MR-PRESSO |
| --- | --- | --- | --- | --- | --- | --- | --- | --- | --- | --- | --- | --- | --- | --- | --- | --- |
|  |  | OR (95%CI) | P  value | Cochran Q  statistics (df) | P  value | OR (95%CI) | P  value | Slope  (95%CI) | P  value | Intercept  (Se) | P  value | Cochran Q  statistics (df) | P  value | OR (95%CI) | P  value | P  value |
| accelerometer-based PA (average acceleration) | 11 | 0.989  (0.942-1.039) | 0.667 | 8.098  (9) | 0.524 | 0.977  (0.914-1.046) | 0.508 | 0.929  (0.793-1.089) | 0.392 | 0.015  (0.019) | 0.437 | 7.430  (8) | 0.491 | 0.973  (0.866-1.094) | 0.662 | 0.598 |
| accelerometer-based PA (acceleration fraction >425 mg) | 4 | 1.616  （1.190-2.193） | 0.002 | 0.513  (3) | 0.916 | 1.789  （0.732-4.372） | 0.202 | 0.080  （5.215e-06-1212.000） | 0.658 | 0.075  (0.121) | 0.601 | 0.135  (2) | 0.935 | 1.840  （0.565-5.987） | 0.386 | 0.898 |
| self-reported moderate-to-vigorous PA | 16 | 0.732  （0.379-1.414） | 0.353 | 17.197  (15) | 0.307 | 0.609  （0.259-1.432） | 0.255 | 0.236  （0.007-7.733） | 0.431 | 0.017  (0.027) | 0.527 | 16.696  (14) | 0.273 | 0.480  （0.116-1.982） | 0.326 | 0.317 |
| self-reported vigorous PA | 4 | 0.993  （0.046-21.630） | 0.997 | 8.122  (3) | 0.044 | 0.333  （0.029-3.851） | 0.379 | 0.011  （7.650e-13-145700000.000） | 0.739 | 0.044  (0.114) | 0.737 | 7.558  (2) | 0.023 | 0.289  （0.018-4.635） | 0.445 | 0.098 |

PA physical activity, IDD intervertebral disc degeneration, LBP low back pain, SNP single nucleotide polymorphism, MR Mendelian randomization, IVW inverse variance weighting, MR-PRESSO MR-Pleiotropy RESidual Sum and Outlier method, WMM weighted median method, Se standard error, df degree of freedom
