# Supplementary material for: Causal association of physical activity with low back pain, intervertebral disc degeneration and sciatica: a two-sample mendelian randomization analysis study
Source: Front Cell Dev Biol. 2023 Nov 9;11:1260001. doi: 10.3389/fcell.2023.1260001 (PMC10665496; doi:10.3389/fcell.2023.1260001)
Supplement: Supplementary file 11 [file DataSheet5.pdf]

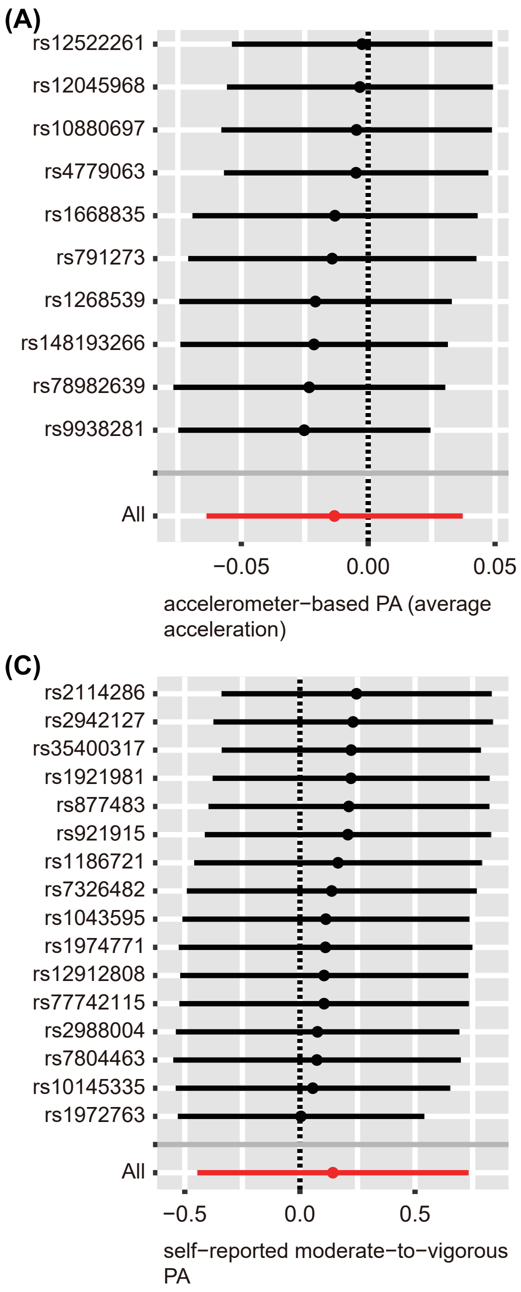

Figure S5: Leave-one-out sensitivity analysis of single SNP for IDD. (A) accelerometer-based PA (average acceleration); (B) accelerometer-based PA (acceleration fraction > 425 milligrams); (C) self-reported vigorous PA; (D) self-reported vigorous PA. Leave-one-out sensitivity analysis is performed to ascertain if a single SNP disproportionately influences an IDD association. Each black point in the forest plot represents the MR analysis (using IVW) excluding that particular SNP. The overall analysis, including all SNPs, is also shown for comparison.
