# Supplementary material for: Causal association of physical activity with low back pain, intervertebral disc degeneration and sciatica: a two-sample mendelian randomization analysis study
Source: Front Cell Dev Biol. 2023 Nov 9;11:1260001. doi: 10.3389/fcell.2023.1260001 (PMC10665496; doi:10.3389/fcell.2023.1260001)
Supplement: Supplementary file 12 [file Table3.DOCX]

Table S3 MR estimates from different methods of assessing the causal effect of PAs on LBP

| PAs | No. of  SNP | IVW | | | | WMM | | MR-Egger | | | | | | Weighted mode | | MR-PRESSO |
| --- | --- | --- | --- | --- | --- | --- | --- | --- | --- | --- | --- | --- | --- | --- | --- | --- |
|  |  | OR (95%CI) | P  value | Cochran Q  statistics (df) | P  value | OR (95%CI) | P  value | Slope  (95%CI) | P  value | Intercept  (Se) | P  value | Cochran Q  statistics (df) | P  value | OR (95%CI) | P  value | P  value |
| accelerometer-based PA (average acceleration) | 11 | 0.945  (0.909-0.986) | 0.005 | 7.618  (9) | 0.573 | 0.947  (0.895-1.003) | 0.063 | 0.896  (0.784-1.024) | 0.146 | 0.013  (0.016) | 0.432 | 6.934  (8) | 0.544 | 0.943  (0.855-1.040) | 0.272 | 0.584 |
| accelerometer-based PA (acceleration fraction >425 mg) | 4 | 1.818  (1.129-2.926) | 0.014 | 1.771  (3) | 0.621 | 1.718  (0.834-3.540) | 0.142 | 9.321  (0.003-299) | 0.642 | -0.040  (0.102) | 0.729 | 1.613  (2) | 0.447 | 2.386  (0.885-6.430) | 0.184 | 0.627 |
| self-reported moderate-to-vigorous PA | 17 | 0.767  (0.397-1.481) | 0.427 | 28.103  (16) | 0.031 | 0.587  (0.286-1.206) | 0.147 | 0.063  (0.002-1.945) | 0.135 | 0.038  (0.026) | 0.167 | 24.634  (15) | 0.055 | 0.486  (0.113-2.093) | 0.347 | 0.038 |
| self-reported vigorous PA | 4 | 0.324  (0.084-1.254) | 0.103 | 2.226  (3) | 0.527 | 0.244  (0.038-1.572) | 0.138 | 0.002  (8.715e-08-49.300) | 0.352 | 0.049  (0.049) | 0.425 | 1.237  (2) | 0.539 | 0.205  (0.015-2.722) | 0.316 | 0.579 |

PA physical activity, IDD intervertebral disc degeneration, LBP low back pain, SNP single nucleotide polymorphism, MR Mendelian randomization, IVW inverse variance weighting, MR-PRESSO MR-Pleiotropy RESidual Sum and Outlier method, WMM weighted median method, Se standard error, df degree of freedom
