# Supplementary material for: Causal association of physical activity with low back pain, intervertebral disc degeneration and sciatica: a two-sample mendelian randomization analysis study
Source: Front Cell Dev Biol. 2023 Nov 9;11:1260001. doi: 10.3389/fcell.2023.1260001 (PMC10665496; doi:10.3389/fcell.2023.1260001)
Supplement: Supplementary file 13 [file Table4.DOCX]

Table S4 MR estimates from different methods of assessing the causal effect of PAs on IDD

| PAs | No. of  SNP | IVW | | | | WMM | | MR-Egger | | | | | | Weighted mode | | MR-PRESSO |
| --- | --- | --- | --- | --- | --- | --- | --- | --- | --- | --- | --- | --- | --- | --- | --- | --- |
|  |  | OR (95%CI) | P  value | Cochran Q  statistics (df) | P  value | OR (95%CI) | P  value | Slope  (95%CI) | P  value | Intercept  (Se) | P  value | Cochran Q  statistics (df) | P  value | OR (95%CI) | P  value | P  value |
| accelerometer-based PA (average acceleration) | 11 | 0.987  （0.938-1.038） | 0.605 | 16.609（9） | 0.055 | 0.991  （0.937-1.048） | 0.741 | 1.070  （0.917-1.250） | 0.416 | -0.020  (0.018) | 0.309 | 14.481（8） | 0.070 | 1.052  （0.937-1.182） | 0.412 | 0.089 |
| accelerometer-based PA (acceleration fraction >425 mg) | 4 | 1.313  （0.975-1.768） | 0.073 | 0.940  （3） | 0.816 | 1.371  （0.745-2.522） | 0.311 | 4.588  （0.004-475） | 0.709 | -0.031  (0.087) | 0.757 | 0.815  （2） | 0.665 | 1.468  （0.642-3.356） | 0.430 | 0.818 |
| self-reported moderate-to-vigorous PA | 16 | 1.154  (0.641-2.078） | 0.633 | 25.725（15） | 0.041 | 1.545  （0.803-2.972) | 0.193 | 1.965  （0.043-89.310） | 0.734 | -0.008  (0.028) | 0.786 | 25.585（14） | 0.029 | 1.770  （0.572-5.485） | 0.338 | 0.050 |
| self-reported vigorous PA | 4 | 0.782  （0.152-4.013） | 0.768 | 4.4078（3） | 0.221 | 1.180  （0.213-6.525） | 0.850 | 0.000  （2.500e-08-0.820） | 0.183 | 0.084  (0.042) | 0.187 | 0.514  （2） | 0.774 | 2.214  （0.124-39.410） | 0.626 | 0.280 |

PA physical activity, IDD intervertebral disc degeneration, LBP low back pain, SNP single nucleotide polymorphism, MR Mendelian randomization, IVW inverse variance weighting, MR-PRESSO MR-Pleiotropy RESidual Sum and Outlier method, WMM weighted median method，Se standard error, df degree of freedom
